# Physiological and molecular mechanisms associated with potato tuber dormancy

**DOI:** 10.1093/jxb/erae182

**Published:** 2024-04-23

**Authors:** Munevver Dogramaci, Emily P Dobry, Evandro A Fortini, Dipayan Sarkar, Dani Eshel, Michael A Campbell

**Affiliations:** Edward T. Schafer Agricultural Research Center, USDA-Agricultural Research Service, Fargo, ND 58102, USA; College of Agricultural Science, Pennsylvania State University, Lake Erie Regional Grape Research and Extension Center, North East, PA 16428, USA; Edward T. Schafer Agricultural Research Center, USDA-Agricultural Research Service, Fargo, ND 58102, USA; Department of Plant Sciences, North Dakota State University, Fargo, ND 58105, USA; Edward T. Schafer Agricultural Research Center, USDA-Agricultural Research Service, Fargo, ND 58102, USA; Department of Postharvest Science, The Volcani Institute, Agricultural Research Organization, Rishon LeZion, Israel; College of Agricultural Science, Pennsylvania State University, Lake Erie Regional Grape Research and Extension Center, North East, PA 16428, USA; University of Western Australia, Australia

**Keywords:** Apical dominance, epigenetics, gene regulation, landraces, oxidative stress, physiological aging, phytohormones, sprout suppressor

## Abstract

Tuber dormancy is an important physiological trait that impacts post-harvest storage and end-use qualities of potatoes. Overall, dormancy regulation of potato tubers is a complex process driven by genetic as well as environmental factors. Elucidation of the molecular and physiological mechanisms that influence different dormancy stages of tubers has wider potato breeding and industry-relevant implications. Therefore, the primary objective of this review is to present current knowledge of the diversity in tuber dormancy traits among wild relatives of potatoes and discuss how genetic and epigenetic factors contribute to tuber dormancy. Advancements in understanding of key physiological mechanisms involved in tuber dormancy regulation, such as apical dominance, phytohormone metabolism, and oxidative stress responses, are also discussed. This review highlights the impacts of common sprout suppressors on the molecular and physiological mechanisms associated with tuber dormancy and other storage qualities. Collectively, the literature suggests that significant changes in expression of genes associated with the cell cycle, phytohormone metabolism, and oxidative stress response influence initiation, maintenance, and termination of dormancy in potato tubers. Commercial sprout suppressors mainly alter the expression of genes associated with the cell cycle and stress responses and suppress sprout growth rather than prolonging tuber dormancy.

## Teaser

This review aims to provide insights into the dormancy-related gene regulation of cultivated and wild-type potato tubers, and the underlying molecular and physiological mechanisms that influence dormancy progression.

## Introduction

Potatoes (*Solanum tuberosum* L.) rank among the top four food crops and are the number one non-grain crop produced globally. Out of >10 000 known potato varieties, 6184 have been documented on the European Cultivated Potato Database (https://www.europotato.org). The International Potato Center (CIP) recorded 4870 accessions of which 4467 are landraces primarily native to the Andean region (CIP, 2024). This genetically diverse crop has cultural, historical, and nutritional significance, and is widely cultivated in different geographic regions around the world. The ability to store the dormant tubers for extended periods has made the potato a staple crop for centuries. Today, the potato continues to be a valuable source of income for growers and nutrition for >1 billion people in the world (Devaux *et al.*, 2021).

Potato tubers are harvested and stored in a dormant stage for year-round use primarily as fresh (26%), frozen (39%), chipped (22%), or dehydrated (7%) products (National Potato Council, 2022). At harvest, cultivated potato tubers are physiologically dormant. During storage, the tubers can be at different dormancy stages including endodormancy, ecodormancy, and paradormancy. Endodormancy, or true dormancy, is regulated by endogenous signals that prevent growth of the meristems even under growth-promoting conditions, while ecodormancy is regulated by exogenous signals (Lang *et al.*, 1987), such that meristems will grow if conditions are conducive. Paradormancy, also known as apical dominance (AD), occurs when growth of lateral buds is inhibited by signals originating from the growing apical bud (Lang *et al.*, 1987). Unlike other plants, the termination of endodormancy and initiation of ecodormancy in potato tubers do not require any specific environmental cues such as chilling hours. Rather, the duration of different dormancy stages is governed by the genetic makeup of cultivars and pre- and post-harvest conditions that contribute to the physiological age of the tubers.

Potato tubers are at greatest nutritional value at harvest, while they are endodormant and high in starch content, and require storage under certain environmental conditions to maintain quality. Otherwise, at the ecodormant stage, metabolic activity and respiration increase, glucose and fructose (reducing sugars) accumulate, glycoalkaloid production increases, and meristematic sprouts begin to develop (Hu *et al.*, 2023). These changes contribute not only to a decrease in fresh weight but also to the ‘sweetening’ of tubers and the potential loss of marketable products. Intricate physiological, molecular, and biochemical mechanisms govern tuber dormancy maintenance. The dormancy trait varies widely among cultivated potato cultivars and wild types. In this review, we summarize the current knowledge of these mechanisms and discuss the impact of common sprout-suppressor treatments on tuber dormancy and overall storage quality.

## Dormancy of *Solanum tuberosum* relatives

The Andean region of South America is considered the origin of potatoes, and hundreds of wild relatives can be found across South and Central America. Studies addressing dormancy in potato landraces or wild relatives have demonstrated a wide range of dormancy periods (Table 1), varying from extremely short to no dormancy for species such as *Solanum chiquidenum* (Horgan *et al.*, 2013) and *Solanum tuberosum* subgroup Phureja (Huamán and Spooner, 2002), to dormancy lasting 8 years or more in some *Solanum jamesii* populations (Bamberg, 2010). Based on these studies, species with longer dormancies tend to originate from semi-arid regions, and those with short or no dormancy tend to originate from high elevation or temperate regions (Table 1). For example, *Solanum kurtzianum* and *Solanum acaule* ssp. *aemulans* are described as being adapted to semi-desert or desert conditions of Argentina (Spooner and Clausen, 1993; Ibañez *et al.*, 2021a), while *S. jamesii* can be found throughout the Southwestern USA (Bamberg, 2010). *Solanum simplicifolium* is native to temperate to humid subtropical regions in Argentina (van den Berg and Spooner, 1992), and *S. chiquidenum* is originally from the highlands of central and northern Peru and mostly distributed in steppe, subtropical steppe, tundra, and desert climates. Given the contradictory trend observed in *S. chiquidenum*, a species with very short dormancy but from a desert-like climate (Bamberg, 2010; Janiszewska *et al.*, 2023), it is evident that environment and habitat can only partially explain the dormancy trait of potato landraces.

**Table 1.** Dormancy duration of wild relatives and landraces of potatoes

Species	Origin	Ploidy	Place of origin	Length of dormancy	Reference
*S. aemulans*	Wild	3×	–	28+ weeks	Janiszewska *et al.* (2023)
*S. chiquidenum*	Wild	2×	Peru	Little to none	Horgan *et al.* (2013)
*S. fendleri*	Wild	4×	Mexico	28+ weeks	Janiszewska *et al.* (2023)
*S. jamesii*	Wild	2×	Southwest USA	8 years	Bamberg (2010)
*S. kurtzianum*	Wild	2×	–	Up to 28 weeks	Janiszewska *et al.* (2023)
*S. multiinterruptum*	Wild	2×	Peru	6+ months	Horgan *et al.* 2013
*S. papita*	Wild	4×	Mexico	28+ weeks	Janiszewska *et al.* (2023)
*S. sandemanii*	Wild	2×	Peru	6+ months	Horgan *et al.* (2013)
*S. simplicifolium* (syn. *S. microdontum*)	Wild	2×	Argentina	Little to none	Janiszewska *et al.* (2023)
*S. tuberosum* subgroup Phureja	Landrace	2×	Venezuela, Bolivia	Little to none	Huamán and Spooner (2002)
*S. tuberosum*×*S. berthaultii*	Landrace×wild	2×	–	Approximately 5.5 months	Hermundstad and Peloquin 1986)
*S. tuberosum*×*S. microdontum*	Landrace×wild	2×	–	Approximately 4 months	Hermundstad and Peloquin (1986)
*S. wittmackii*	Wild	2×	Peru	6+ months	Horgan *et al.* (2013)

Variability of experimental methods within these studies limits the ability to draw comparisons or formulate new hypotheses for future studies of landraces and wild types. In the landmark study by Bamberg (2010), tubers of *S. jamesii* collected from different locations of the Southwestern USA remained dormant for >8 years at 5 °C. The prolonged dormancy of this species might be due to the natural adaptation to the harsh environmental conditions of this region. Supporting extended dormancy as a potential survival mechanism against unfavorable environmental conditions, Horgan *et al.* (2013) observed that tubers of accessions of some wild potato species (e.g. *Solanum sandemanii*, *Solanum multiinterruptum*, and *Solanum wittmacki*) with longer dormancy (~6 months) exhibited higher resistance against potato tuber moth (*Phthorimaea operculella*). Hermundstad and Peloquin (1986) reported varied dormancy length within and between clones of the same crosses. For example, within *S. tuberosum×Solanum microdontum*, dormancy ranged from 58 d to 165 d, while dormancy lasted for 130–190 d in crosses of *S. tuberosum×Solanum berthaultii*. In these studies, endodormancy length was not specifically measured, nor were characteristics of tuber quality, gene expression, or phytohormone changes described. Such information would significantly increase our understanding of molecular mechanisms regulating dormancy in potato species and potentially contribute to enhancing dormancy characteristics of potato cultivars.

## Genetic and epigenetic regulation associated with tuber dormancy

The length of tuber dormancy is dependent on both the genotype and environmental conditions during tuber bulking and maturation (Sonnewald, 2001), and can range from 1 to 15 weeks after harvest if tubers are kept under growth-conducive conditions (Wiltshire and Cobb, 1996). However, there is a lack of comprehensive evaluation of dormancy length in many commercial cultivars as well as of the genetics that contribute to dormancy. More detailed analysis of genes that control dormancy has been conducted in interspecific hybrids. Crosses between *S. tuberosum*×*Solanum chacoense* and *Solanum phureja* demonstrated a relationship between 22 markers and tuber dormancy, with markers on chromosome 7 being major contributors to long dormancy (Freyre *et al*., 1994). Genetic studies have shown at least nine quantitative trait loci (QTLs) involved with dormancy in crosses between *S. tuberosum* and *S. berthaultii* (van den Berg *et al.*, 1996). The backcross to *S. berthaultii* resulted in recessive gene contributions to long dormancy. QTLs controlling dormancy length, as well as abscisic acid (ABA) levels, have also been demonstrated in interspecific crosses and backcrosses of potato (Simko *et al*., 1997; Bisognin *et al*., 2018). A recent study identified the significance of a gene associated with a large effect QTL on chromosome 3, TERMINAL FLOWER 1/CENTRORADIALIS (*StCEN)*, in transgenic tubers of *S. tuberosum* cv. Désirée (Morris *et al*., 2019). Expression levels of the gene were closely related to sprout development and ABA concentrations (Morris *et al*., 2019), though its exact role in dormancy regulation remains to be elucidated.

**Fig. 1.**
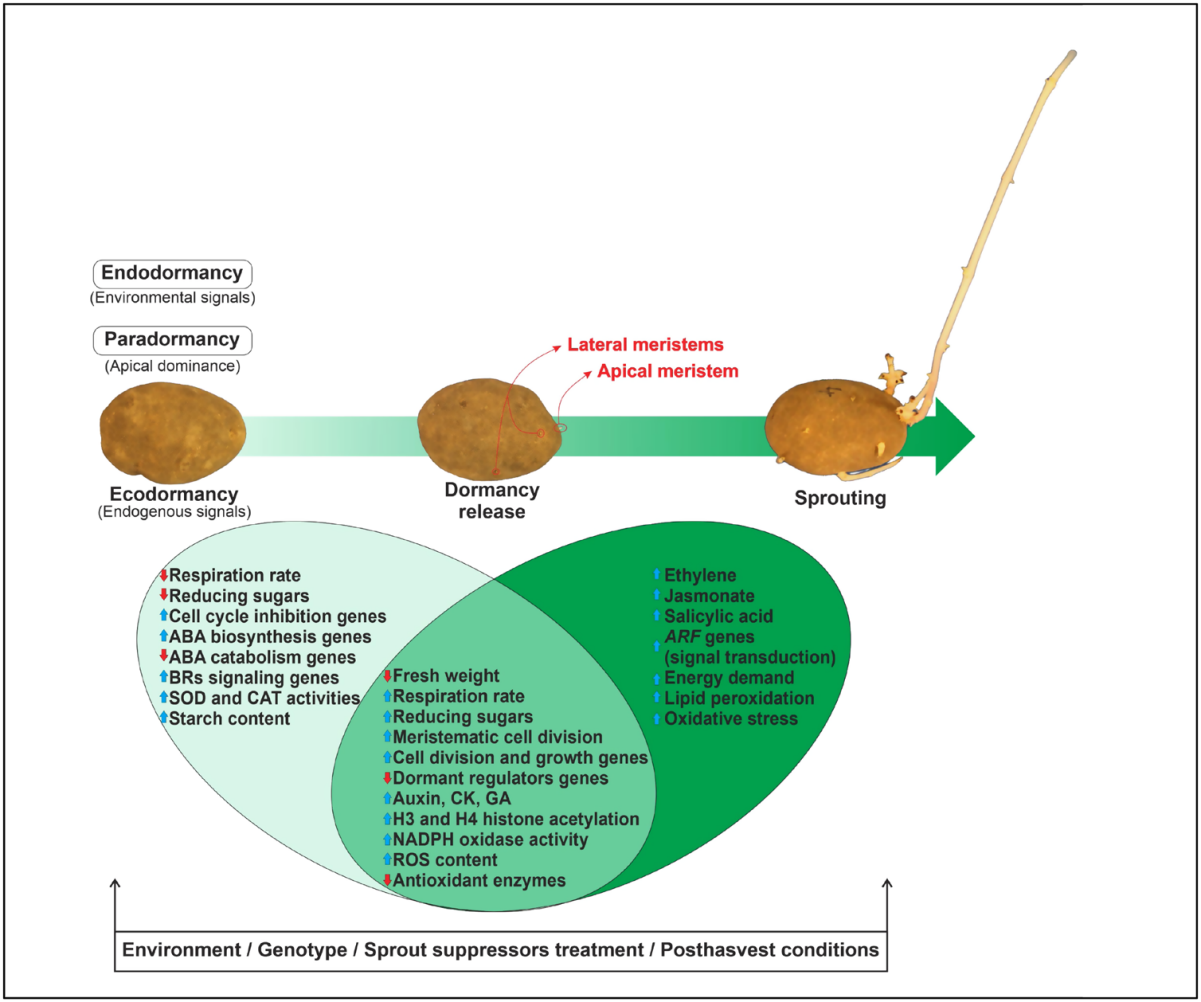
Key physiological and molecular mechanisms impacting tuber dormancy and sprouting.

Dormancy in potato is a polygenic trait and is regulated by a complex network of genetic processes (Wang *et al.*, 2022; Cai *et al*., 2023; Zheng *et al*., 2023). Transcriptional analyses have revealed numerous genes and networks involved in regulating tuber dormancy. Changes in abundance of transcripts and proteins related to cell cycle, phytohormone regulation, stress response, carbohydrate metabolism, or energy production were observed in tuber meristems of different potato cultivars between different dormancy stages (endodormant to ecodormant) and between the dormant and non-dormant (sprouting) stage (Fig. 1) (Campbell *et al.*, 2008, 2014, 2020; Sonnewald and Sonnewald, 2014; Liu *et al.*, 2015a) (full gene names are given in Table 2). Cell division is generally arrested in the pre-mitotic phase during the dormant stage of the tubers (Suttle, 2004). Campbell *et al.* (2012) observed increased abundance of transcripts involved in cell cycle inhibition (e.g. cyclin-dependent kinase inhibitor *KRP1* and *KRP2*) in sprout suppressor-treated tuber meristems of ecodormant cv. Russet Burbank. During dormancy termination, increased expression of transcripts related to cell division and growth [e.g. delta (24)-sterol reductase-like (*GhFe1*)] was quantified in tuber meristems (Campbell *et al.*, 2008). Increased transcript abundance of D-type cyclin *CYCD3* was also observed during tuber dormancy termination and with exogenous cytokinin treatment, while transcript abundance of the *AGL* transcription factor gene (a putative dormancy regulator) decreased (Campbell *et al.*, 2014) (Table 2).

**Table 2.** Genetic regulation of dormancy in potato tubers

Gene specification	Key function	Expression	Dormancy state	Cultivar	Sprout suppressor treatment	Reference
Ovule/fiber elongation protein (*GhFe1*)	Cell cycle regulation	Up-regulation	Non-dormant (2–5 mm of sprouting after 150 d in cold storage)	Russett Burbank	Bromoethane (BE)	Campbell *et al.* (2008)
Kip related protein 1 (*KRP1*)	Cell cycle regulation	Up-regulation	Endodormant	Russett Burbank	DMN	Campbell *et al.* (2012)
Kip related protein 2 (*KRP2*)	Cell cycle regulation	Up-regulation	Endodormant	Russett Burbank	DMN	Campbell *et al.* (2012)
D-type cyclin (*CYCD3*)	Cell cycle regulation	Up-regulation	Non-dormant (≥2 mm of sprouting)	Russett Burbank and Atlantic	Cytokinin agonist 1-(α-ethylbenzyl)-3-nitroguanidine (NG)	Campbell *et al.* (2014)
AGAMOUS-like MADS-box-like (*AGL*)	Cell cycle regulation	Down-regulation	Non-dormant (≥2 mm of sprouting)	Russett Burbank and Atlantic	NG	Campbell *et al.* (2014)
Regulator of chromosome condensation 1 (*RCC1*)	Cell cycle regulation	Up-regulation	Endodormant	Longshu 3	Untreated	Liu *et al.* (2015a)
Histone gene (*H1*)	Cell cycle regulation	Up-regulation	Endodormant	Longshu 3	Untreated	Liu *et al.* (2015a)
Histone gene (*H3*)	Cell cycle regulation	Up-regulation	Endodormant	Longshu 3	Untreated	Liu *et al.* (2015a)
Histone gene (*H4*)	Cell cycle regulation	Up-regulation	Endodormant	Longshu 3	Untreated	Liu *et al.* (2015a)
Histone chaperone anti-silencing function 1 (Asf1)	Cell cycle regulation	Up-regulation	Endodormant	Longshu 3	Untreated	Liu *et al.* (2015a)
Cell division cycle (*CDC6*)	Cell cycle regulation	Up-regulation	Endodormant	Longshu 3	Untreated	Liu *et al.* (2015a)
Cell division cycle (*CDC45*)	Cell cycle regulation	Up-regulation	Endodormant	Longshu 3	Untreated	Liu *et al.* (2015a)
Cyclin A	Cell cycle regulation	Up-regulation	Endodormant	Longshu 3	Untreated	Liu *et al.* (2015a)
Cyclin B	Cell cycle regulation	Up-regulation	Endodormant	Longshu 3	Untreated	Liu *et al.* (2015a)
Cyclin C	Cell cycle regulation	Up-regulation	Endodormant	Longshu 3	Untreated	Liu *et al.* (2015a)
Cyclin D	Cell cycle regulation	Up-regulation	Endodormant	Longshu 3	Untreated	Liu *et al.* (2015a)
Ran GTPase activating protein 2 (*RGA2*)	Stress response	Up-regulation	Non-dormant (after initiation of sprouting)	Longshu 3	Untreated	Liu *et al.* (2015a)
Thaumatin-like protein (*TLP*)	Stress response	Up-regulation	Non-dormant (after initiation of sprouting)	Longshu 3	Untreated	Liu *et al.* (2015a)
Pathogenesis related protein (*PR*)	Stress response	Up-regulation	Non-dormant (after initiation of sprouting)	Longshu 3	Untreated	Liu *et al.* (2015a)
Heat shock protein (*HSP*)	Stress response	Up-regulation	Non-dormant (after initiation of sprouting)	Longshu 3	Untreated	Liu *et al.* (2015a)
NAD kinase (*NADK*)	Stress response	Up-regulation	Non-dormant (after initiation of sprouting)	Longshu 3	Untreated	Liu *et al.* (2015a)
Auxin response factor (*ARF*)	Auxin signaling	Down-regulation	Endodormant and ecodormant	La Chipper	DMN	Campbell *et al.* (2020)
Auxin response factor 6 (*ARF6*)	Auxin signaling	Undetected expression	Endodormant	Desirée	DMN	Faivre-Rampant *et al*. (2004)
Indole 3-acetic acid inducible 15 (*IAA15*)	Auxin signaling	Differently expressed	After initiation of sprouting	subsp. *andigena*	Giberellin (GA_3_), 6-benzyladenine (CK), or GA_3_+CK	Zhang *et al*. (2023)
Indole 3-acetic acid inducible 22 (*IAA22*)	Auxin signaling	Differently expressed	After initiation of sprouting	subsp. *andigena*	GA_3_, CK, or GA_3_+CK	Zhang *et al*. (2023)
Small auxin up-regulated RNA 50 (*SAUR50*)	Auxin signaling	Differently expressed	After initiation of sprouting	subsp. *andigena*	GA_3_, CK, or GA_3_+CK	Zhang *et al*. (2023)
Indole-3-acetic acid-induced protein (ARG7) located close to the QTL region at 40.48 Mb	Auxin signaling		Dormancy release	Diploid wild species–*S. chacoense* and *S. berthaultii*	Untreated	Bisognin *et al.* (2018)
Indole-3-acetic acid-amido synthetase (GH3.6) located close to the QTL region at 47.67 Mb	Auxin catabolism		Dormancy release	Diploid wild species–*S. chacoense* and *S. berthaultii*	Untreated	Bisognin *et al.* (2018)
Cytochrome P450 monoxygenase, family 735, subfamily A (*CYP735A*)	Zeatin biosynthesis	Differently expressed	After initiation of sprouting	subsp. *andigena*	GA_3_, CK or GA_3_+CK	Zhang *et al*. (2023)
*Cis*-zeatin *O*-glucosyltransfer- ase (*CISZOG*)	Zeatin biosynthesis	Differently expressed	After initiation of sprouting	subsp. *andigena*	GA_3_, CK, or GA_3_+CK	Zhang *et al*. (2023)
UDP-glycosyltransferase 85A1 (*UGT85A1*)	Zeatin biosynthesis	Differently expressed	After initiation of sprouting	subsp. *andigena*	GA_3_, CK, or GA_3_+CK	Zhang *et al*. (2023)
Pneumolysin 4 (*PLY4*)	ABA regulation	Differently expressed	After initiation of sprouting	subsp. *andigena*	GA_3_, CK, or GA_3_+CK	Zhang *et al*. (2023)
9-*cis*-epoxy-carotenoid dioxygenase 1 (*StNCED1*)	ABA regulation	Down-regulation	Non-dormant (after initiation of sprouting)	Russett Burbank	BE	Destefano-Beltran *et al.* (2006)
9-*cis*-epoxy-carotenoid dioxygenase 2 (*StNCED2*)	ABA regulation	Down-regulation	Non-dormant (after initiation of sprouting)	Russett Burbank	BE	Destefano-Beltran *et al.* (2006)
Cytochrome P450, family 707, subfamily a, polypeptide 1 (*StCYP707A1*)	ABA regulation	Up-regulation	Non-dormant (after initiation of sprouting)	Russet Burbank; Longshu 3; VR808 and Shelford	BE; untreated; 1-methylcyclopropene (1-MCP) and ethylene	Destefano-Beltran *et al.* (2006); Liu *et al.* (2015a); Tosetti *et al*. (2021)
Responsive to dehydration-induced (RD22)	ABA regulation	Down-regulation	Non-dormant (2–5 mm of sprouting after 150 d in cold storage)	Russett Burbank	BE	Campbell *et al.* (2008)
Tubby like protein (TUBBY) genes located at 57.48 Mb on chromosome 3 and 52.53 Mb on chromosome 7	ABA signaling		Dormancy release	Diploid wild species-S. chacoense & S. berthaultii	Untreated	Bisognin *et al.* (2018)
Cytokinin oxidase 1 (*CKX1*)	Cytokinin regulation	Up-regulation	Dormant meristem in tissue culture medium	Solara	GA3 and CK	Hartmann *et al*. (2011)
Teosinte branched1/Cincinnata/proliferating 15 (*StTCP15*)	GA signaling	Up-regulation	Non-dormant after 30 d of storage at room temperature	Desiree	Untreated	Wang *et al.* (2022)
Repressor of the gibberellin 4 (*RGA4*)	GA signaling	Down-regulation	Dormant tubers	Holland no. 7	Untreated	Xiong *et al*. (2022)
Gibberellin 2-oxidase 2 (GA2ox2) located close to the QTL region on chromosome 7 at 51.9 Mb	GA catabolism		Dormancy release	Diploid wild species–*S. chacoense* and *S. berthaultii*	Untreated	Bisognin *et al.* (2018)
Ethylene receptor homolog (*ERH*)	Ethylene regulation	Up-regulation (under ethylene) or downregulation (1-MCP)	Dormant tubers	VR808 and Shelford	1-MCP and continue ethylene (CE)	Tosetti *et al.* (2021)
1-Aminocyclopropane-1-carboxylate oxidase (*ACO*)	Ethylene regulation	Up-regulation (under ethylene) or downregulation (1-MCP)	Dormant tubers	VR808 and Shelford	1-MCP and CE	Tosetti *et al.* (2021)
1-Aminocyclopropane-1- carboxylate synthase (*ACS*)	Ethylene regulation	Down-regulation	Dormant tubers	VR808 and Shelford	1-MCP and CE	Tosetti *et al.* (2021)
Ethylene response factor 1 (ERF1) on chromosome 6 at 45.88 Mb	Ethylene regulation		Dormancy release	Diploid wild species–*S. chacoense* and *S. berthaultii*	Untreated	Bisognin *et al.* (2018)
Lipoxygenase (*LOX*)	Jasmonate biosynthesis	Down-regulation	Endodormant	Longshu 3	Untreated	Liu *et al.* (2015a)
WRKY transaction factors	Jasmonate biosynthesis	Up-regulation	After termination of endodormancy	Russett Burbank	DMN	Campbell and D’Annibale (2016)
Snakin-2 (*StSN2*)	Brassinosteroid signaling	Up-regulation	Dormant tubers	Chuanyu 10	Untreated	Liu *et al.* (2023)
Brassinosteroid-insensitive 2 (*StBIN2*)	Brassinosteroid signaling	Up-regulation	Dormant tubers	Chuanyu 10	Untreated	Liu *et al.* (2023)
Brassinazole resistant 1 (*StBZR1*)	Brassinosteroid signaling	Inhibition	Dormant tubers	Chuanyu 10	Untreated	Liu *et al.* (2023)
Sucrose phosphate synthase (*SPS*)	Carbohydrate metabolism	Up-regulation	Sprouting initiation	Chuanyu 10	Untreated	Cai *et al.* (2023)
ADP-ribosylation factors (*ARF*)	Vesicular traffic regulation and signal transduction	Up-regulation	Sprouting (2 mm bud)	Favorita	Untreated	Liu *et al.* (2012)

Liu *et al.* (2015a) reported 932 transcripts unique to the endodormant state in potato cv. Longshu 3, while a unique set of 629 transcripts was expressed during dormancy release, and 550 transcripts were specific to sprouting tubers. In the same study, transcripts associated with auxin, brassinosteroids (BRs), cytokinins, gibberellic acid (GA), cell division, and cell cycle regulation were found to increase at the ‘dormancy release’ stage, while transcripts associated with ethylene, jasmonic acid (JA), and salicylic acid (SA) increased during dormancy termination and sprouting. An increase in transcript abundance of genes (e.g. WRKY-type transcription factor, wound-inducible PR4 protein) associated with stress following treatment with the sprout suppressor 1,4-dimethylnaphthalene (DMN) was observed (Campbell *et al.*, 2010, 2012, 2020; Campbell and D’Annibale, 2016), suggesting that stress responses may alter dormancy release or prevent sprouting based on the level of stress. In eukaryotic systems, cell cycle checkpoints are influenced by stress signals, which can result in arrest of cell division (Bertoli *et al*., 2013). This may suggest that stress responses enhance the termination of endodormancy. However, elevated stress, such as that induced by a chemical sprout suppressor, would suppress cell division in tuber meristems once endodormancy is exited.

Previous research provided some insights on the changes in hormone-related gene expression associated with tuber dormancy stages and termination (Fig. 1). In a study with tubers of cv. La Chipper, Campbell *et al.* (2020) reported that during all stages of dormancy and storage, transcripts involved in auxin signaling [e.g. ADP-ribosylation factor (ARF)] were down-regulated by sprout-suppressor treatment. Previously, Suttle *et al.* (2014) noticed that although *CKX* gene expression and enzyme activity were present in tuber buds throughout the dormancy period, they do not appear to play a significant role in the regulation of cytokinin content during dormancy progression. However, Hartmann *et al.* (2011) reported that *CKX1*-expressing tubers remained dormant for a longer period and delayed sprouting up to 8 weeks. Hartmann *et al.* (2011) also investigated the ectopic expression of *GA 20-oxidase* in potato, which resulted in diminished dormancy and extended sprout development, indicating the function of GA in dormancy release. Wang *et al.* (2022) reported that *StTCP15* interacts with *StGID1* to reduce expression of DELLA proteins and regulate GA_3_ signaling to promote sprouting (Table 2). *StTCP15* promotes sprouting through regulation of the ABA:GA balance, as overexpression of *StTCP15* reduced the ABA:GA ratio (Wang *et al.*, 2022). The application of GA, either alone or with cytokinin, resulted in significant changes in expression of genes related to ABA (*4-PLY4*), auxin (*IAA15*, *IAA22*, *SAUR50*), and zeatin biosynthesis (*CYP735A*, *CISZOG*, and *UGT85A1*), indicating the pivotal role of these genes in dormancy release (Zhang *et al.*, 2023).

Decreased expression of *NCED1/2* and increased expression of *CYP707A1*, which are involved in ABA biosynthesis and catabolism, respectively, were reported during tuber dormancy termination (Destefano-Beltran *et al*., 2006). A decreased abundance of transcripts associated with the ABA-inducible RD22 protein was observed in tuber during dormancy termination (Campbell *et al.*, 2008). During storage, ethylene treatment induces the expression of *ACO*, while 1-methylcyclopropene (1-MCP), an ethylene-binding inhibitor, inhibits *ACO* and *ACS* (Tosetti *et al*., 2021) (Table 2). A decrease in transcripts encoding *LOX*, which is involved in JA biosynthesis, was observed in dormant tubers (Liu *et al.*, 2015a). Extended tuber dormancy was observed with overexpression of *StSN2* and *StBIN2*, which negatively regulate BR signaling, while expression of *StBZR1* was inhibited (Liu *et al*., 2023). Increased expression of the transcription factor gene *ARF*, which is involved in vesicular transport and signal transduction, was observed during sprouting of tubers (Liu *et al.*, 2012). The authors observed that expression of *ARF* is also associated with hormone signaling and carbohydrate metabolism (Liu *et al.*, 2012). Collectively, these findings suggest that genetic factors that regulate phytohormone metabolism play significant roles in dormancy regulation and interact with other physiological processes such as stress responses and carbohydrate metabolism.

Along with genetic factors, epigenetic factors also contribute to potato tuber dormancy and sprouting. Epigenetic mechanisms, such as DNA methylation, histone modifications, and RNA-mediated alterations, play a crucial role in activating or repressing genes associated with dormancy regulation processes. When activated during dormancy establishment, epigenetic markers require deactivation to allow growth to resume (Rohde and Bhalerao, 2007). Epigenetics plays a key role in regulating potato tuber development, as studies have found links between the levels of epigenetic markers with the stolon-to-tuber transition and tuberization (Kondhare *et al.*, 2018, 2021; Tiwari *et al*., 2021). Indeed, changes in DNA methylation levels impact plant resistance to stresses such as osmotic stress (Harding, 1994) and drought (Bi *et al*., 2022). This mechanism is also likely to be vital in maintaining the genetic variability and phenotypic plasticity of wild potato plants that thrive in diverse environments (Ibañez *et al*., 2021b).

Changes in storage conditions can also impact the epigenetic modifications, potentially affecting dormancy length and establishment (Xiong *et al*., 2022). Research has demonstrated that exposing tubers to light during storage can result in genomic demethylation and decreased expression of *RGA4*, an inhibitor of GA signaling (Xiong *et al*., 2022). Similarly, in apple (*Malus domestica*) buds, changes in histone methylation patterns induced GA and auxin signaling, leading to dormancy break in response to seasonal temperature fluctuations (Chen *et al*., 2022). These findings indicate that environmental signals can activate or deactivate epigenetic markers, directly influencing dormancy stages. Epigenetic markers also influence dormancy initiation and establishment. In potato suspension cell cultures, variations in histone acetylation occurred after modifications of the overall degree of DNA methylation. These processes seem to be associated with cell division and growth (Law and Suttle, 2005). Furthermore, the demethylation of cytosine sequences in DNA impacts gene expression regulation during tuber development and dormancy break (Law and Suttle, 2003).

Histone modifications play a key role in onset and termination of dormancy. During dormancy release in potato meristems, a natural increase in acetylation of histones H3 and H4 was observed (Law and Suttle, 2004). These authors also found that this change can be induced by treating the tubers with bromoethane (BE), a potent dormancy-breaking inducer. Campbell *et al.* (2020) reported that sprout-suppressor treatment impacted DNA methylation/chromatin modifications. During dormancy release, RNA-mediated modifications have emerged as fundamental epigenetic regulators. As potato tubers sprout, a reconfiguration in the expression patterns of many long non-coding RNAs was observed (Hou *et al*., 2017). This reconfiguration may be related to coordinating various biological processes that occur during the transition from dormancy to growth reinitiation in tuber meristems. However, there is still a lack of evidence on the specific biological function of these RNAs in activating or silencing genes related to metabolic pathways, as well as their role in resuming growth in potato tubers (Hou *et al*., 2017).

## Physiological aging, apical dominance, and tuber dormancy

The physiological aging of tubers, which happens due to internal biochemical changes, impacts dormancy, sprouting, and future crop performance (Knowles and Knowles, 2016). Unlike many other organisms, the chronological age and physiological age of potato tubers vary due to internal and external factors. Aging in biological systems can be determined through changes in molecular and metabolic activities that lead to loss of cellular viability or tissue damage over time, such as senescence (Coleman, 2000). In potato, cultivar (genetic) differences and temperature during cultivation and post-harvest storage contribute significantly to physiological aging and alter the duration and progression of post-harvest developmental stages of tubers (Eshel and Teper-Bamnolker, 2012). Loss of AD during storage is considered one of the indicators of a tuber’s physiological age (Eshel and Teper-Bamnolker, 2012). Delaplace *et al.* (2008) developed a physiological age index based on the correspondence values of dormancy duration, loss of AD, and sprouting using two potato cultivars. Still, the accuracy of such methods is often disputed due to numerous factors that can potentially confound the results. Thus, the utility of biological indicators of physiological age and biomarkers associated with AD needs to be examined thoroughly.

AD in potato tubers refers to the apical bud’s influence in controlling lateral bud growth. This concept is analogous to the AD exerted by the shoot tip in various other species (Dun *et al*., 2006; Leyser, 2009; Beveridge *et al.*, 2023). Tuber sprouting typically starts from the tuber apical meristem (TAM), which is positioned opposite to the connection point between the tuber and stolon. Usually, one eye or sprout becomes dominant and suppresses growth of the other eyes. Auxin was considered as a major regulator of AD through alteration of cytokinin, ABA, and strigolactone metabolism in woody and other plant species (Beveridge *et al.*, 2023). However, a recent study has also highlighted a potential auxin-independent phytohormone regulation and sugar release influencing AD in pea (Cao *et al.*, 2023). Despite potato tubers serving as a model system for investigating metabolic processes related to endodormancy release, there have been limited efforts to establish a connection between dormancy release and AD (Michener, 1942; Holmes *et al*., 1970; Kumar and Knowles, 1993; Salam *et al.*, 2017). Michener (1942) showed that when the intact tuber grows after dormancy release, one or more apical buds grow, but lateral buds usually do not. If, however, lateral buds and apical buds are excised, both will grow at the same time. Moreover, in non-dormant tubers, any first-growing, large bud usually inhibits growth of the later growing, smaller ones (Michener, 1942).

**Fig. 2.**
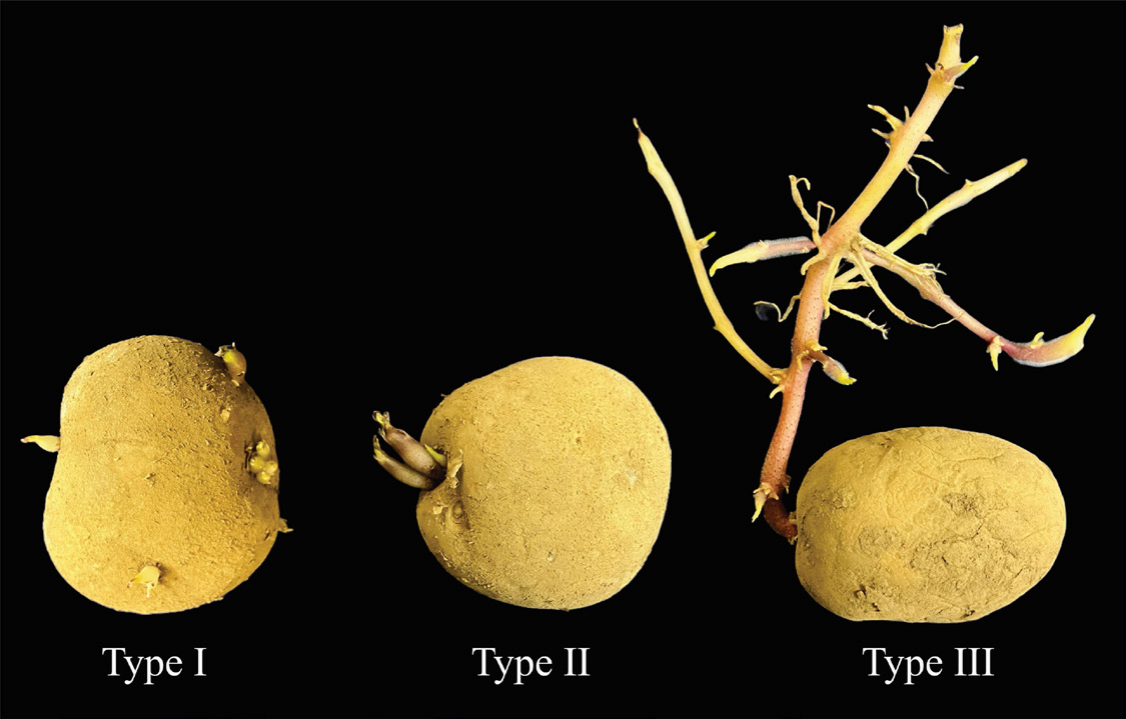
Three types of apical dominance (AD) loss in stored potato tubers. Loss of dominance over basipetal buds (type I), axillary buds within the same eye (type II), and excessive branching of the developing apical meristem (type III).

Teper-Bamnolker *et al.* (2012) observed three main types of AD loss in stored potatoes: loss of dominance of apical buds over those situated more basipetally on the tuber (‘type I’); loss of dominance of the primary bud in any given eye over the subtending axillary buds within the same eye (‘type II’); and loss of dominance of the developing TAM over more basal buds, meaning that side stems do not emerge from the base of the sprout as in type II (‘type III’) (Fig. 2). Loss of type I dominance results in behavior like a typical stem. However, the developing apical bud only suppresses mature or dormancy-released buds. When the apical bud is removed, it triggers early sprouting of all other mature buds within the same tuber. After 30, 60, and 90 d in cold storage, on average, one, two, and nine buds sprouted, respectively (Teper-Bamnolker *et al.*, 2012), suggesting the need for each bud to reach maturity and autonomous dormancy release before the TAM controls it. Cline (1997) distinguished between initiation of axillary bud growth and subsequent axillary shoot elongation, which may be under the control of different hormone factors, as shown by Hartmann *et al.* (2011). Removing a lateral meristem complex or wounding between buds did not impact AD or sprouting rate (Teper-Bamnolker *et al.*, 2012; Buskila *et al*., 2016). These experiments emphasize the importance of TAM presence and viability in controlling lateral bud meristem growth before sprouting is observed.

Irregular sprouting behavior is one of the earliest morphophysiological signs that a tuber has been subjected to stress. For example, the phytotoxic chemical BE shortens the natural dormancy period from 2–4 months to ~10 d (Destefano-Beltran *et al*., 2006; Campbell *et al.*, 2008; Alexopoulos *et al*., 2009). Campbell *et al.* (2008) observed that transcript profiles during BE-induced cessation of dormancy are similar to those observed in natural dormancy release, suggesting that both follow a similar biological pattern during this transition. Teper-Bamnolker *et al.* (2012) showed that BE application induces early sprouting in freshly harvested ‘Nicola’ and ‘Désirée’ tubers, as well as loss of AD. Buds surrounding the apical buds tended to grow faster than those in more distant tuber segments. Loss of type I AD as a result of BE treatment was followed by loss of type III dominance, expressed as excessive branching of the growing shoots (Teper-Bamnolker *et al.*, 2012). Teper-Bamnolker *et al.* (2010) showed that very low doses of the sprout suppressor R-carvone also induced early sprouting and loss of AD. While high doses of this suppressor damaged apical meristem cell membranes, no such damage was detected when the sprout-inducing low dose was used, suggesting a signaling effect. At both doses, the result was loss of all types of AD, when the tuber sprouted, leading to a bush-like pattern of growing buds (Teper-Bamnolker *et al.*, 2010).

The mode of action of phytotoxic chemicals in inducing dormancy release and altering AD is poorly understood. Programmed cell death (PCD) in the potato TAM was proposed as one of the mechanisms regulating AD (Teper-Bamnolker *et al.*, 2012; T. Liu *et al.*, 2017). Hallmarks of PCD were identified in the TAM during normal growth, which were more extensive when AD was lost following either extended cold storage or BE treatment. Hallmarks included DNA fragmentation, induced gene expression of vacuolar processing enzyme 1 (VPE1), and elevated VPE activity (Teper-Bamnolker *et al.*, 2012, 2017, 2020). Treatment of tubers with BE and then a VPE inhibitor induced faster growth and AD recovery in detached and non-detached apical buds, respectively, suggesting that PCD is associated with weakening of tuber AD, allowing early sprouting of mature lateral buds (Teper-Bamnolker *et al.*, 2012).

Cold storage is the main tool used worldwide to delay sprouting of stored tubers. Long cold storage is one of the main factors inducing early dormancy release and loss of AD, correlated with sweetening of the parenchyma (Salam *et al.*, 2017; Danieli *et al*., 2023; Teper-Bamnolker *et al.*, 2023). Cold temperature alters sugar metabolism and influences AD and dormancy release (Sonnewald and Sonnewald, 2014). When the tuber is exposed to cool temperatures during dormancy, the number of sprouting buds after dormancy release increases with time of exposure. In other words, an increase in the number of weeks of exposure to cool temperatures reduces AD (Hartmans and Van Loon, 1987; Fauconnier *et al*., 2002; Struik, 2007).

The number of stems emerging from the soil is affected by mother tuber genetics, and growing and storage conditions (Danieli *et al*., 2018; Dean *et al*., 2018). Dormancy release, AD, and stem branching are sequential events that are probably affected by hormonal regulation and the available energy in the tuber’s storage tissue (Salam *et al.*, 2021; Danieli *et al*., 2023; Roumeliotis *et al*., 2023). However, these can be altered by numerous abiotic stresses, including storage temperature and chemical sprout suppressors. These factors have been shown to induce PCD in the potato TAM. Decapitation experiments performed with sprouting tubers have shown the importance of keeping TAM cells viable for maintenance of AD (Teper-Bamnolker *et al.*, 2012; Buskila *et al.*, 2016).

## Phytohormone regulation in dormancy and sprouting of potato tubers

As highlighted in the genetic regulation section of this review, it is becoming evident that endogenous phytohormones play an essential role in tuber dormancy control (Suttle, 2007; Campbell *et al.*, 2010; Sonnewald and Sonnewald, 2014; Bisognin *et al*., 2018; Peivastegan *et al*., 2019; Tai *et al*., 2020; Tosetti *et al*., 2021). Both ABA and ethylene are required for dormancy induction, but only ABA is required to maintain tuber dormancy. The content of both *cis*- and *trans*-cytokinins increases immediately prior to sprouting, while increases in GA and auxin content appear to be more closely related to subsequent sprout growth. Suttle *et al.* (2012) reported that chemical manipulation of ABA metabolism and content had no effect on tuber dormancy duration, suggesting that dormancy release may be controlled by an increase in growth-promoting cytokinin rather than reduced ABA levels. However, down-regulation of ABA-responsive genes was observed during tuber dormancy release (Campbell *et al.*, 2008; Bisognin *et al.*, 2018). Wang *et al.* (2020) observed that exogenous nitric oxide treatment promotes expression of genes involved in ABA catabolism (*StCYP707A1*, which encodes ABA 8'-hydroxylase), while inhibiting expression of a gene involved in ABA biosynthesis (*StNCED1*, encoding 9-*cis*-epoxycarotenoid dioxygenase) and subsequently induces sprouting in potato tubers.

Studies elucidated that tubers exhibit increases in cytokinin content and significant changes in abundance of transcripts involved in cytokinin signaling during dormancy progression (Suttle, 1998, 2007; Suttle and Banowetz, 2000), but cytokinin metabolism or cytokinin oxidase activity in tuber meristems does not change substantially during dormancy progression, suggesting that cytokinin catabolism does not play a major role in the regulation of cytokinin content or activity (Suttle *et al.*, 2014). Clearly, hormone content represents the dynamic balance between the rates of biosynthesis and catabolism, and the interplay among phytohormones modulates their precise abundance for regulating growth and developmental processes, or in response to varying environmental conditions. Higher expression of *StTCP15* (TEOSINTE BRANCHED1/CYLOIDEA/PCF15), which is associated with the GA signaling pathway, was observed during termination of potato tuber dormancy (Che *et al.*, 2022). Increased expression of the *ARF1* gene during dormancy breaking was also reported in potato tuber (Liu *et al.*, 2012). The role of GA in dormancy release and elongated sprout growth was reported by Hartmann *et al.* (2011). Additionally, other phytohormones such as JA, SA, and BRs also influence dormancy and sprouting of tubers either through their direct biological functions or through interactions with other phytohormones such as cytokinin, GA, ABA, auxin, and ethylene (Peivastegan *et al*., 2019; Hu *et al*., 2023). Liu *et al*. (2023) reported that *StBIN2* (BRASSINOSTEROID-INSENSITIVE 2) influences tuber dormancy by regulating ABA and BR signaling. Increased expression of BR synthesis genes such as *BRI1* (BRASSINOSTEROID-INSENSITIVE 1) was observed during dormancy release and particularly in potato varieties with a short dormancy period (Zou *et al.*, 2017). However, most dormancy-related studies in potato have focused on the content and role of specific hormone(s) and often integrated with expression of a selected set of genes. Consequently, such studies do not always capture the complex molecular mechanisms underlying dormancy. Thus, further studies are needed implementing a holistic approach to develop a comprehensive understanding of the phytohormone metabolism and regulation controlling tuber dormancy progression and sprouting.

## Critical role of oxidative stress and antioxidants in potato tuber dormancy and sprouting

Reactive oxygen species (ROS), antioxidant networks, and associated signaling play important roles in tuber dormancy regulation (Liu *et al*., 2015b; Beauvieux *et al*., 2018). The controlled accumulation of ROS, including hydrogen peroxide (H_2_O_2_), superoxide, and hydroxyl radicals, contributes to tuber dormancy termination and sprouting processes. In contrast, antioxidant enzymes, such as superoxide dismutase (SOD) and catalase (CAT), regulate cellular ROS concentration, playing a fundamental role in modulating tuber dormancy (Liu *et al*., 2015b).

Overall, an increase in ROS generation during long-term storage and with the aging of potato tubers is associated with dormancy termination and sprouting. Due to the increased energy demand during sprouting, there is a rise in the activity of ATPases, resulting in elevated levels of lipid peroxidation and oxidative stress (Kumar and Knowles, 1996). Increased NADPH oxidase activity, which indicates higher oxidative stress and influences GA metabolism, potentially leads to tuber dormancy termination (B. Liu *et al.*, 2017). Therefore, metabolic processes that induce oxidative stress seem to trigger the transition from dormancy to sprouting, involving complex interactions between ROS and plant hormones.

Several genes involved in dormancy regulation and physiological aging of tubers are associated with oxidative stress (Liu *et al.*, 2012). Overexpression of the transcription factor genes *StDREB1* and *Vitis vinifera WRKYGQK* (*VvWRKY2*) influences cellular ROS concentration through enhanced activity of antioxidant enzymes in tubers (Chiab *et al*., 2023). Similarly, increased expression of the *StSN2* gene, which contributed to a significant reduction in the accumulation of H_2_O_2_ and enhanced activity of SOD and CAT, was observed during prolonged tuber dormancy (Deng *et al*., 2021). On the other hand, the expression of *Respiratory burst oxidase homolog* genes (*StRbohA and StRbohB*; involved in generation of ROS) correlated with dormancy release, supporting the idea that the increased activity of NADPH oxidases leads to an increase in the ROS content and subsequent development of sprouts (B. Liu *et al.*, 2017).

Several reports indicated that the activity of antioxidant enzymes (e.g. CAT) decreases significantly in tubers throughout storage, with a subsequent increase in the accumulation of H_2_O_2_ and oxidative stress, leading to dormancy breaking (Rojas-Beltran *et al*., 2000; Bajji *et al*., 2007; Liu *et al.*, 2012). Potatoes treated with the sprout suppressors menthol and eugenol showed a lower content of polyphenol oxidases (PPOs) and peroxidases, which influences the processing quality of the potato (Santos *et al*., 2020). Likewise, tubers treated with garlic essential oil as a sprout suppressor resulted in an up-regulation of the proteins associated with SOD and PPO, which may have contributed to the quenching of ROS and subsequently extended dormancy duration (Li *et al*., 2022). Wet storage of potatoes has also been reported to promote prolonged dormancy and less sprouting (Peivastegan *et al*., 2019) by inducing a hypoxic stress condition in the tubers, which may have led to transcriptional regulation of genes associated with hormones (e.g. ABA and SA) and antioxidant mechanisms, resulting in a decrease of oxidative stress. Therefore, ROS play a fundamental role in regulating tuber dormancy stages, and controlled manipulation of ROS balance could potentially be used to improve post-harvest storage qualities. However, how the changes in balance between ROS and antioxidant networks influence dormancy needs to be investigated during different stages of dormancy and with sprout-suppressor treatments. Overall, a better understanding of the physiological processes associated with oxidative stress has significant implications for the post-harvest management of potatoes, opening up possibilities for novel strategies to manage dormancy and maintain tuber quality.

## Artificial dormancy regulation through sprout-suppressor treatments

Following harvest and skin set, sprout suppression is important to maintain tuber quality during long-term commercial storage. Sprout suppression can be achieved without the use of chemical treatment by storing at near-freezing temperatures. Seed tubers, which are used for planting rather than consumption, can be stored for long periods at temperatures of 2–4 °C without concern for cold-induced sweetening (CIS). This condition occurs when there is a restriction of glycolysis and starch is converted to sucrose, which is then hydrolyzed into reducing sugars (Dixon and Rees, 1980; Sonnewald, 2001). CIS and accumulation of reducing sugars are associated with darker frying color for products such as chips and French fries (Marquez and Añon, 1986), and thus reduced commercial desirability. For this reason, it is recommended that tubers stored for consumption are kept at warmer temperatures, generally ranging from 4 °C to 7 °C for the fresh market, and from 7 °C to 11 °C for chipping and frying tubers. However, tubers stored at warmer temperatures have increased rates of sprouting. To reduce the effects of sprouting on tuber quality, sprout-suppressing treatments are commonly employed.

There are several sprout suppression options currently available to growers such as chlorpropham (CIPC), ethylene, maleic hydrazide, DMN, and essential oils. These have varying costs, availability, environmental and health concerns, and periods of efficacy, and may require one or multiple applications, or uninterrupted exposure. Their use also induces differing long-term quality characteristics such as reducing sugar accumulation and glycoalkaloid content. For growers, consideration of cost and quality is of high importance as they affect the price of the produce. It is important to note that none of these sprout suppressors induces or prolongs tuber dormancy; rather, they inhibit sprouting by reducing respiration and metabolic activity. CIPC and essential oils, in particular, induce burned, necrotic meristems that cannot resume growth after treatment, effectively killing the growing tissue. For the purposes of this review, discussion will be limited to CIPC, DMN, and ethylene as they are the most used sprout suppressors.

**Fig. 3.**
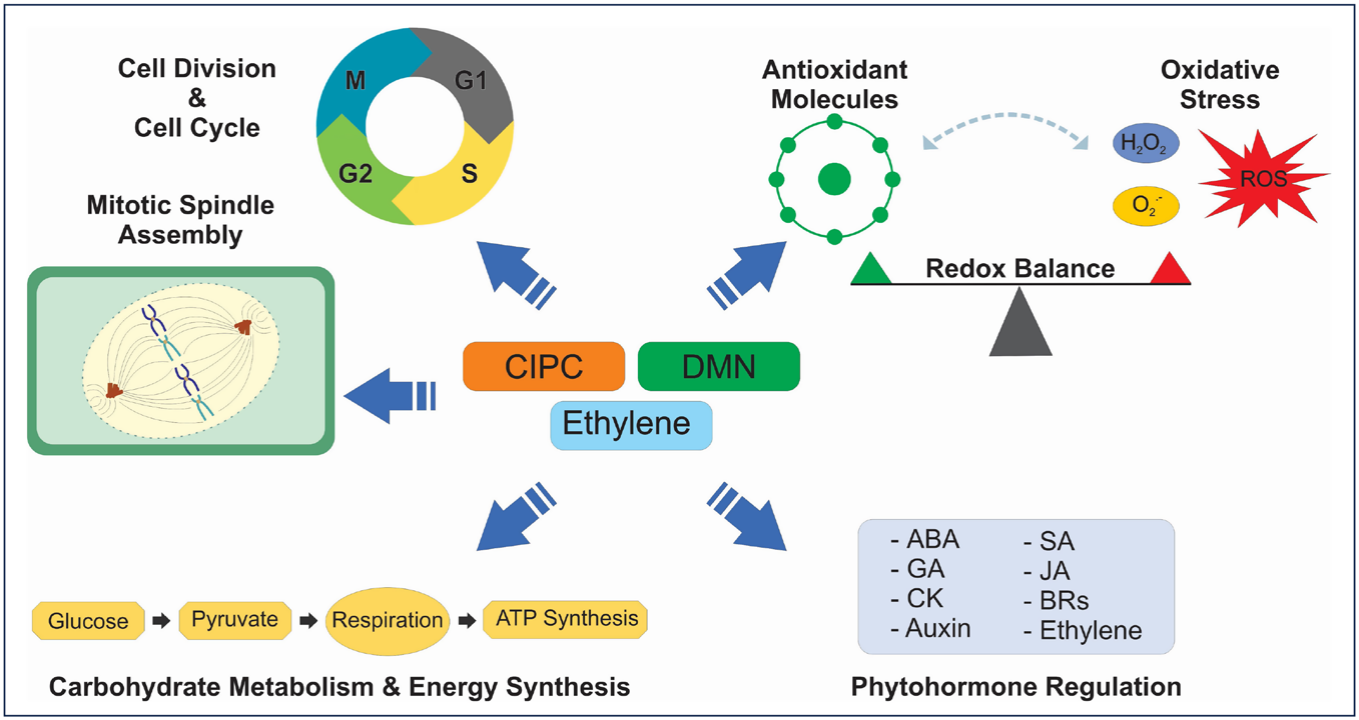
Potential modulatory role of sprout suppressor treatments (CIPC, DMN, and ethylene) on key physiological and biochemical regulation associated with the cell cycle and cell division, oxidative stress, carbohydrate metabolism and energy synthesis, and phytohormones.

CIPC is the most widely utilized sprout suppressor due in part to its comparatively low cost, the low number of required applications, and its demonstrated efficacy. The compound suppresses sprout growth by inhibiting mitotic spindle formation and orientation during cell division (Hepler and Jackson, 1969; Eleftheriou and Bekiari, 2000) (Fig. 3). For this reason, CIPC should not be applied before wound healing or curing, as it inhibits periderm formation (Reeve *et al*., 1963; Leach, 1978). Inhibition of wound healing is associated with increased weight loss and greater incidence of rot, and poor tuber quality (Leach, 1978; Knowles *et al*., 1982). The mode of action also precludes it from use on seed tubers, as it results in diminished sprouting (Kim *et al.*, 1972), lower emergence rate, lower yield, and altered tuber size (Frazier and Olsen, 2015).

The ability of CIPC to maintain tuber quality for extended periods is also well documented. Tubers treated with CIPC experience less starch degradation and reducing sugar accumulation (Khurana *et al*., 1985), and reduction in respiration rates (Boe *et al*., 1974), weight loss (Blenkinsop *et al*., 2002), and glycoalkaloid content (Wu and Salunkhe, 1977). Most importantly, CIPC has been demonstrated to maintain chipping and frying color quality standards even after several months of storage (Blenkinsop *et al*., 2002; Krause *et al*., 2023). Recent research has raised concerns regarding its continued use, however, due to chemical residues in storage. These studies demonstrated that CIPC accumulates in concrete floors with repeated application and residues remained for decades after the last application occurred (Douglas *et al*., 2018). Chemical residue is of concern because CIPC degrades into several secondary products, many of which are considered toxic. Residues of CIPC and its main derivative 3CA have been detected on peels of commercially stored potatoes (Mohammed *et al*., 2015) and as bound and free analytes in processed potatoes (Göckener *et al*., 2020). Together, these regulatory concerns led to the recent decision by the European Union to ban the use of CIPC (Visse-Mansiaux *et al.*, 2021a) and push for acceptable alternative methods of sprout suppression.

DMN is a naturally occurring compound in potatoes (Meigh *et al*., 1973), which has been found to be an effective sprout suppressor (Beveridge *et al.*, 1981). While the mode of action of DMN is presently unknown, evidence suggests that it suppresses sprout growth through disruption of cell cycle progression and induction of stress responses (Fig. 3) (Campbell *et al.*, 2010, 2012, 2020; Campbell and D’Annibale, 2016). For this reason, DMN should be applied after wound healing has occurred. Effects of DMN are not permanent, and multiple applications are needed during long-term storage. Due to its temporary sprout suppression effect, DMN can be used on seed tubers (Beveridge *et al.*, 1981) up to 30 d prior to planting (1,4Group, 2018).

As with CIPC, DMN has a demonstrated capacity to maintain tuber quality parameters for extended periods in storage. Tubers treated with DMN experience less starch degradation and reducing sugar accumulation than control tubers, and often exhibit levels of both that are comparable with CIPC-treated tubers (Yang *et al*., 1999; Visse-Mansiaux *et al.*, 2021b; Krause *et al*., 2023). DMN also suppresses the number of sprouts per tuber, which is comparable with CIPC (Nyankanga *et al*., 2018). DMN-treated tubers have been shown to maintain chipping (Mendonça Neto *et al*., 2022) and frying color quality (Krause *et al*., 2023) following extended periods in storage. DMN can also protect against rotting and infection in storage (Santos *et al.*, 2023). This evidence supports DMN as a commercially acceptable alternative to CIPC.

To indeed be a commercial alternative, DMN must demonstrate a reduced risk to human health and the environment. A report by the European Food Safety Authority (EFSA) assessed the potential environmental fate of DMN, finding that it did not form metabolites or accumulate in sediment, had a low risk of long-range transport in the air, and determining it to be readily biodegradable (European Food Safety Authority, 2013). According to the same report, the compound is rapidly metabolized and excreted by mammals, has low toxicity, and does not demonstrate any carcinogenic or genotoxic activity. Further, the EFSA later reported that risk to consumers’ health is low and post-harvest treatment of potatoes does not result in toxic exposure levels of DMN or its metabolites in the diet (European Food Safety Authority, 2023). While more research is needed, these reports suggest that use of DMN poses less risk to human and environmental health than CIPC.

Ethylene is another effective sprout suppressor that naturally occurs within potatoes (Rylski *et al*., 1974; Prange *et al*., 1998). Since 2002, this phytohormone has been registered for use as a potato sprout suppressor in several nations (Daniels-Lake *et al*., 2011). While the mode of action of sprout suppression by ethylene remains unknown, tubers treated with ethylene have exhibited increased expression of genes related to defense and stress responses (Yang *et al*., 2020; Tosetti *et al*., 2021), probably signaling unfavorable growing conditions. Ethylene is a known regulator of senescence processes of plant tissues through crosstalk with major phytohormones such as ABA, auxin, cytokinin, and GA (Iqbal *et al.*, 2017). Ethylene signaling pathways also interact with WRKY transcription factors and influence senescence through cooperative actions with JA signaling (Koyama, 2014; Kim *et al.*, 2015). However, the crosstalk of ethylene with other phytohormones in the context of potato tuber dormancy and sprouting needs to be thoroughly investigated.

It is essential to note that depending on the concentration and duration of exposure, exogenous ethylene can either accelerate or delay sprouting (Rylski *et al*., 1974; Foukaraki *et al*., 2016a, b). This may be a result of rapid catabolism of the dormancy-promoting hormone ABA (Suttle *et al.*, 2013; Tosetti *et al*., 2021), potentially reducing it below the threshold required to maintain a dormant state (Fig. 3). Unlike CIPC and DMN that may require multiple applications, tubers must be continuously exposed to ethylene to maintain sprout suppression. Also, because different ethylene exposure regimes can shorten dormancy and tubers resume sprouting once exposure has ceased, this hormone can be safely used on seed tubers to break dormancy and promote uniform sprouting.

Ethylene exposure reduced levels of glycoalkaloids such as α-tomatine in tomatoes (Iijima *et al*., 2009; Itkin *et al*., 2011), which may be the case in potatoes. Ethylene supplementation can also increase the content of reducing sugars in potato tubers (Daniels-Lake *et al*., 2005), which negatively affects processing quality. Foukaraki *et al.* (2016a) observed that ethylene exposure resulted in increased accumulation of reducing sugars, and this accumulation was higher when exposure began immediately after harvest than when it began after dormancy break. These findings support the numerous studies that observed darker fry color following ethylene treatment (Prange *et al*., 1998, 2005; Daniels-Lake *et al*., 2005, 2011). There is evidence that the color is dose dependent and varies by cultivar (Daniels-Lake *et al*., 2011); thus, agronomically significant cultivars should be evaluated for color response prior to utilizing ethylene in commercial storage.

Adding an ethylene-binding inhibitor (1-MCP) with ethylene treatment eliminated ethylene-induced sugar accumulation (Foukaraki *et al*., 2016a; Tosetti *et al*., 2021), and enhanced the sprout-suppressing effect of ethylene (Tosetti *et al*., 2021). The frequency of ethylene treatment (continuous or intermittent), the duration of effectiveness in delaying early sprouting, and its impact on gene expression and consequent changes in carbohydrate metabolism have not yet been completely clarified (Foukaraki *et al*., 2016a, b; Alamar *et al*., 2017). Tosetti *et al.* (2021) investigated the effects of continuous ethylene and 1-MCP treatment on the transcriptome as well as ABA and ABA metabolites in potato tubers, which showed that ethylene had a strong enhancer effect on parenchymatic ABA catabolism that caused a significant decrease in ABA levels and a transient rise in the ABA catabolite phaseic acid. Tosetti *et al.* (2021) further emphasized the lack of a link between exogenous ethylene treatment and expression of a previously reported gene (*StCEN*) that was shown to have a clear effect on manipulation of potato sprout growth (Morris *et al.*, 2019). While these reports provide insights for molecular mechanisms associated with ethylene-induced sprout growth suppression, there is much to learn on the regulatory mechanisms controlling sprout growth inhibition as a result of ethylene supplementation.

To date, there are no reports addressing potential environmental or health concerns surrounding the use of ethylene for sprout suppression. However, given the transient effects of ethylene following cessation of exposure, and its application in gaseous form, it is unlikely to accumulate in storage. This also makes it unlikely to accumulate and remain in the tuber at levels which might be toxic for consumption. More research is needed to determine its potential influence on concentrations of other toxic compounds.

## Conclusion

Tuber dormancy is affected by both internal and external factors. A prolonged dormancy period in some wild relatives of potatoes is potentially part of their natural adaptation to extreme habitat conditions, and diversity in dormancy duration offers opportunities to integrate them in breeding strategies for developing new varieties with prolonged dormancy traits. Furthermore, insights on genetic and epigenetic regulation associated with cell division, phytohormone metabolism, and oxidative stress response might help to identify suitable biomarkers that can also be utilized in advanced breeding and molecular strategies to improve varieties with desirable dormancy traits. Overall, maintenance of longer dormancy duration requires cessation of the cell cycle, higher levels of ABA content, and suppression of ROS. Post-harvest storage strategies and novel sprout-suppressor treatments might be needed to modulate these molecular and physiological control points to influence tuber dormancy. Future investigations with different potato cultivars/accessions, varying storage conditions, and with commercial and experimental sprout-suppressor treatments will shed more light on the dormancy regulation of potato tubers and how it can be modulated for optimum storage and end-use qualities.
